# Comparative Transcriptome Analysis of *Bombyx mori* (*Lepidoptera*) Larval Midgut Response to BmNPV in Susceptible and Near-Isogenic Resistant Strains

**DOI:** 10.1371/journal.pone.0155341

**Published:** 2016-05-11

**Authors:** Xue-Yang Wang, Hai-Zhong Yu, Lei Geng, Jia-Ping Xu, Dong Yu, Shang-Zhi Zhang, Yan Ma, Dong-Qiong Fei

**Affiliations:** School of Life Sciences, Anhui Agricultural University, Hefei, People’s Republic of China; Institute of Plant Physiology and Ecology, CHINA

## Abstract

*Bombyx mori* nucleopolyhedrovirus (BmNPV) is one of the primary pathogens causing severe economic losses in sericulture. However, the molecular mechanism of silkworm resistance to BmNPV remains largely unknown. Here, the recurrent parent P50 (susceptible strain) and the near-isogenic line BC9 (resistance strain) were used in a comparative transcriptome study examining the response to infection with BmNPV. A total of 14,300 unigenes were obtained from two different resistant strains; of these, 869 differentially expressed genes (DEGs) were identified after comparing the four transcriptomes. Many DEGs associated with protein metabolism, cytoskeleton, and apoptosis may be involved in the host response to BmNPV infection. Moreover, some immunity related genes were also altered following BmNPV infection. Specifically, after removing genetic background and individual immune stress response genes, 22 genes were found to be potentially involved in repressing BmNPV infection. These genes were related to transport, virus replication, intracellular innate immune, and apoptosis. Our study provided an overview of the molecular mechanism of silkworm resistance to BmNPV infection and laid a foundation for controlling BmNPV in the future.

## Introduction

The silkworm, *Bombyx mori* L. (Lepidoptera: Bombycidae) has been domesticated for production of cocoons for more than 5000 years. Silkworm rearing and the silk industry still play an important role in China, India and many other developing countries. *B*. *mori* is also a good model for the study of insect genetics and immunology [1–4]. *Bombyx mori* nucleopolyhedrovirus (BmNPV) is the principal silkworm pathogen and causes serious economic losses in sericulture every year. Among numerous silkworm strains, most are susceptible to BmNPV infection, although a few strains exhibit resistance [5]. The heredity of silkworm resistance against BmNPV infection is a relatively complicated process because resistance is controlled both by major dominant genes and multiple genes of micro-effect [6].

A series of studies have made significant progress in understanding silkworm resistance against BmNPV infection. Xu et al. reported that *Bms3a* was potentially involved in resistance to BmNPV infection [7, 8]. *B*. *mori* lipase-1, serine protease-2 and alkaline trypsin protein extracted from the digestive juice of larvae midguts showed strong antiviral activity *in vitro* [9–11]. Using comparative proteomics, arginine kinase was found to be involved in the antiviral process of different resistant strains of silkworm [12]. In our laboratory, a total of 12 proteins that are potentially involved in viral infection were identified using one- and two-dimensional electrophoresis followed by virus overlay assays. These proteins could be categorized into the following groups: endocytosis, intracellular transportation, and host responses [13]. Immune responses were found to be synergistically regulated by the Toll, Janus kinase/signal transducers and activators of the transcription (JAK/STAT) and immune deficiency (IMD) pathways, which could act as an important defense against exogenous pathogenic infection in conjunction with subsistent pathogen recognition receptors and response proteins [14–18]. However, the molecular mechanisms of silkworm resistance to BmNPV infection are still not fully elucidated.

In recent years, the high-throughput nature of next generation sequencing (NGS), using platforms such as Illumina HiSeq^TM^ 2500 have provided fascinating opportunities in the life sciences and dramatically improved the efficiency and speed of gene discovery, especially in the research of host cell responses to exogenous pathogenic infection [19]. For example, Hu et al. obtained numerous differentially expressed genes (DEGs) involved in metabolism, immunity, and inflammatory responses in *Microtus fortis* following infection with *Schistosoma japonicum* based on comparative transcriptome analysis [20]. Diege et al. examined different fish tissues infected with salmon anemia virus (ISAV) using high-throughput transcriptomics and found a strong correlation between functional modules and viral-segment transcription [16]. NGS technology was also used to explore the molecular mechanism of silkworm resistance against exogenous pathogens. Kolliopoulou et al. reported that several genes related to physical barriers, immune response, proteolytic/metabolic enzymes, heat-shock proteins, and hormonal signaling were possibly involved in silkworm resistance against *Bombyx mori* cytoplasmic polyhedrosis virus (BmCPV) infection; although these genes might be induced by the virus in order to increase infectivity [21]. Additionally, several candidate genes, such as *BmEts*, *BmToll10-3* and *Hsp20-1*, have been identified in the initial stage of BmNPV infection by analyzing the global transcriptional profile of silkworm cell lines and heads following BmNPV infection [22, 23].

In order to gain a global view of the molecular changes in silkworms during BmNPV infection, we selected near-isogenic line BC9 and recurrent parent P50 for transcriptome sequencing. Through comparative analysis of the transcriptomes from these two strains, a total of 869 DEGs were obtained, which included many genes potentially related to BmNPV-resistance. Our results may provide some reliable evidence to clarify the BmNPV-resistance molecular mechanism in silkworm.

## Materials and Methods

### Virus and Silkworm

BmNPV (T3 strain) was maintained in the Key Laboratory of Sericulture, Anhui Agricultural University, Hefei, China. The virus was obtained from the hemolymph of infected larvae and purified by repeated and differential centrifugation according to the protocol developed by Rahman et al. [24]. The concentration of the virus (OB/mL) was determined by hemocytometer.

The recurrent parent P50 (susceptible strain), the donor parent A35 (resistant strain) and the near-isogenic line BC9 were maintained in our laboratory. The near-isogenic line was constructed according to the protocol used by Yao et al. [6]. In brief, the recurrent parent P50 was crossed to the donor parent A35; progeny were repeatedly backcrossed with the recurrent parent for nine generations and each progeny was screened by BmNPV.

The first three instar larvae were reared on a fresh artificial diet at 26±1°C, 75±5% relative humidity, and a 12 hours day/night cycle. The rearing temperature for the last two instars was reduced to 24±1°C; other conditions were unchanged. On the first day of fifth instar, all larvae were starved for 24 hours and then fed with 5 μL BmNPV suspended in sterile water (1.0×10^5^ OB/mL) per larva orally; the control group was treated with sterile water. BmNPV occlusion bodies (OB) began fast proliferation at approximately 24 hours post inoculation (hpi) [25]; thus, this time was considered optimal for sample collection. Silkworm larvae were dissected and the midgut tissues were removed and then washed in PBS (137 mM NaCl, 2.7 mM KCl, 4.3 mM Na_2_HPO_4_, and 1.4 mM KH_2_PO_4_, pH 7.4) prepared with diethy pyrocarbonate (DEPC) (Sangon, China) treated H_2_O. Thirty larvae midguts were mixed together to minimize individual genetic differences. Samples were flash-frozen in liquid nitrogen and pulverized, and 100 mg of sample were added directly into a RNAase free microcentrifuge tube containing 1.0 mL TRIzol Reagent (Invitrogen, USA) and stored at -80°C for later use.

### Silkworm strain resistance level bioassays

The level of silkworm resistance to BmNPV was tested following the protocol developed by Cheng et al. [26]. The fourth instar larvae were inoculated with BmNPV at different concentrations; inoculations were conducted in triplicate. The level of silkworm resistance was calculated using IBM SPSS Statistics 20 (IBM, USA).

### RNA extraction

The silkworm midguts dissolved in TRIzol Reagent were homogenized. Total RNA of midguts were extracted according to the manufacturer’s protocol. Concentrations were quantified using a NanoDrop 2000 spectrophotometer (Thermo Scientific, USA). The purity of all RNA samples were assessed at an absorbance ratio of A_260/280_ and A_260/230_, and the integrity of the RNA was confirmed by 1% agarose gel electrophoresis.

### Library construction, Illumina sequencing and read assembly

Fragment interruption, cDNA synthesis, addition of adapters, PCR amplification and RNA-Seq were performed by Beijing BioMarker Technologies (Beijing, China). The standard Illumina methods and protocols were adopted to prepare and sequence the cDNA libraries. NEBNext Poly(A) mRNA Magnetic Isolation Module (NEB, USA) was used to enrich mRNA, and then the cDNA library was constructed using the NEBNext mRNA Library Prep Master Mix Set for Illumina (NEB, USA) and NEBNext Multiplex Oligos for Illumina (NEB, USA). The size of the library insert fragments was determined by 1.8% agarose gel electrophoresis, and the fragments were quantified using a Library Quantification Kit/Illumina GA Universal (Kapa, USA). Suitable fragments were selected as templates and sequenced on an Illumina HiSeq^TM^ 2500 using paired-end technology. Three biological replicates were used to minimize sample differences.

In order to obtain clean and high-quality reads for sequence assembly, the raw reads were filtered by removing adaptor sequences, low-quality sequences (reads with ambiguous bases ‘N’) and reads with 10% > Q < 20% bases [27, 28]. The Trinity assemble program was used to assemble the clean reads into contigs, which covered more full-length transcripts through a broad range of expression levels [29]. The resultant contigs were added to transcripts based on paired-end information. The longest transcript from alternative splicing transcripts was selected as the unigene. These unigenes were combined to produce the final assembly and used for annotation.

### Functional annotation

To annotate unigenes, different sequences were searched by BLASTx against the NCBI non-redundant protein (nr) database and other databases, including Swiss-Prot protein database, the Kyoto Encyclopedia of Genes and Genomes (KEGG) and the Cluster of Orthologous Groups (COG) database. Gene Ontology (GO) annotations, including molecular functions, biological processes, and cellular components, were obtained using the Blast2GO program (https://www.blast2go.com/) [30, 31]. All searches were performed with an *E-*value < 10^−5^. Fragments per kilobase of transcript per million fragments mapped (FPKM) was calculated to represent the expression abundance of the unigenes [32]. FPKM may reflect the molar concentration of a transcript by normalizing for RNA length and for the total read number.

### Differentially expressed genes (DEGs) analysis

After normalizing genes expression levels, DEGs were obtained by pair-wise comparison of the four transcriptome libraries using IDEG6 software [33]. An FPKM fold change of ≥ 1.2 or ≤ 0.83 between two libraries was defined as the reference standard, with the Benjamini–Hochberg false discovery rate (FDR < 0.01) used to adjust the p-values.

### Real-time quantitative PCR analysis

In order to validate the results from our transcriptome sequencing analysis, the relative expression levels of 15 randomly selected genes were confirmed by reverse transcription quantitative PCR (RT-qPCR). Additionally, 9 genes with well reported previously were selected to further validate the genes of interest that might be involved in BmNPV resistance. All the Primers are listed in Table 1. RT-qPCR reactions were prepared with the SYBR Premix Ex Taq^TM^ Kit (TaKaRa), following the manufacturer’s instruction. Reactions were carried out in Bio-Rad CFX96TM Real-Time System (Bio-Rad, USA). The thermal cycling profile consisted of an initial denaturation at 95°C for 30 s, 40 cycles at 95°C for 5 s, and 60°C for 30 s. All samples were performed in triplicate. Relative expression levels were calculated using the 2^-△△Ct^ method following the protocol of Livak et al. [34]. In this study, *B*. *mori* ribosomal protein s3 (*BmRPS3*) gene was used as a reference gene. Statistical analysis was conducted using the SPSS software (IBM, www.ibm.com).

**Table 1:** Primers used in RT-qPCR for validation of DEGs.

Gene ID	Forward Primer	Reverse primer
BGIBMGA004355	5' GAGAGTTCCCTTGTCGCTTGTG 3'	5' CTCGCAGTTTGCTTTCGTAGTG 3'
BGIBMGA001480	5' CGAGATGGTGTGCTATGGGAAT 3'	5' CGATTTGTCGTCGTTTCAGGA 3'
NewGene_4242	5' TGTCAGTCCCCTCGTTGCTTG 3'	5' CGTTGTGGATTGGTCATCATTCA 3'
BGIBMGA013757	5' TAATGGGTTCCCTTGTGTAATGGT 3'	5' TGTTAGCGAGGTAGTGCCTTTCA 3'
BGIBMGA010400	5' ACGGCTTGGGCGTTGCTATT 3'	5' CGGTTATCTCCGCTTCTGTGCT 3'
BGIBMGA011531	5' CGCCTTCAGAAACACAAGTCGT 3'	5' CGTATCCCATCCTGCTGGTAAC 3'
BGIBMGA013756	5' CCGTGGGTGCTCCCTATGAT 3'	5' GCGGGGTTTGACGAAATGAA 3'
BGIBMGA007540	5' TTCCCATCGTCAAAGAACTCG 3'	5' TTAGCGGTAATAGCGGCAGA 3'
BGIBMGA005673	5' TGCTATGGGTGTGGGTGAAATC 3'	5' GGAAGGCGTCAAAACGAATG 3'
BGIBMGA000680	5' CCATACTACTGCGGTGTCGGTG 3'	5' CATTCCAATCTTGAACGGGCTTA 3'
BGIBMGA010023	5' TGTTTCTCTGGAGCCTTCTACCG 3'	5' GAGTGTTTCCCCAACCGATGA 3'
BGIBMGA000583	5' GAGCAGGGTGATTAGAGCGTTGT 3'	5' CCCACTCGTGAGGAGCGGTA 3'
BGIBMGA010811	5' CAACCCAACAGTTATCGCCG 3'	5' GCTCTCGCATCATCTCCACAT 3'
BGIBMGA007377	5' GAGTCACAACCAGAACCATTGCTAT 3'	5' TACCGACGAAGGAGAGGAACG 3'
BGIBMGA001320	5' GACCCTGAAAACCATCACCCA 3'	5' CGGAGTCGCCGAAGCAAGT 3'
BGIBMGA004002	5' CACTGAGCCAATCGTGCCCT 3'	5' GACTTCATCGGACTCGTCAACAA 3'
BGIBMGA004121	5' CCATCAATAGTCCCAGCACCG 3'	5' CGCTCACAGCACCACCGTCT 3'
BGIBMGA008867	5' AATGGATTCAGGTTTGGACGC 3'	5' CCGACGCTTCTCTTCTTGTTCT 3'
BGIBMGA014369	5' CGGCTCCCTATGGCTTTGG 3'	5' CGGTCAGGACAGTCATCTTCGTG 3'
BGIBMGA004869	5' GTATTGTTGTTTGTTGTGGCAGC 3'	5' CGTGGACTTCGGGATTCTCA 3'
BGIBMGA006775	5' GTGCCAGAGGTTCATCCAGC 3'	5' TGATACAGCCATAGCGGTTCC 3'
BGIBMGA010062	5' GACGGCAACCTCCACAAGC 3'	5' CAGGGGCACCCAAGTCAGTA 3'
BGIBMGA006518	5' CCACGAAGCCAGAAGGATTGT 3'	5' AAGACGGAGGTAGCGAAGGAG 3'
BGIBMGA009012	5' TACAACGATGTGCCCAGTGC 3'	5' CCTTCTTGAGTCCAGCAAATACC 3'
BGIBMGA009319 (*BmRPS3*)	5' CGATTCAACATTCCAGAGCA 3'	5' GAACACCATAGCAAGCACGAC 3'

## Results

### BmNPV infectivity in different silkworm strains

The LC_50_ value was used to evaluate the resistant level of silkworm to BmNPV infection. The LC_50_ value of A35 was approximately 26-fold greater than that of BC9 and over 500-fold greater than that of P50. The value of BC9 was 23-fold greater than that of P50 (Table 2).

**Table 2:** The LC_50_ value of *B*. *mori* larvae infected with BmNPV.

Strains	LC_50_	95% fiducial limits	
	(OB/mL)	Lower	Upper
**BC9**	2.27×10^6^	4.58×10^5^	1.74×10^7^
**A35[26]**	5.90×10^7^	2.14×10^7^	3.22×10^8^
**P50[26]**	1.03×10^5^	3.96×10^4^	2.24×10^5^

### Overview of the silkworm transcriptome

**Fig 1:**
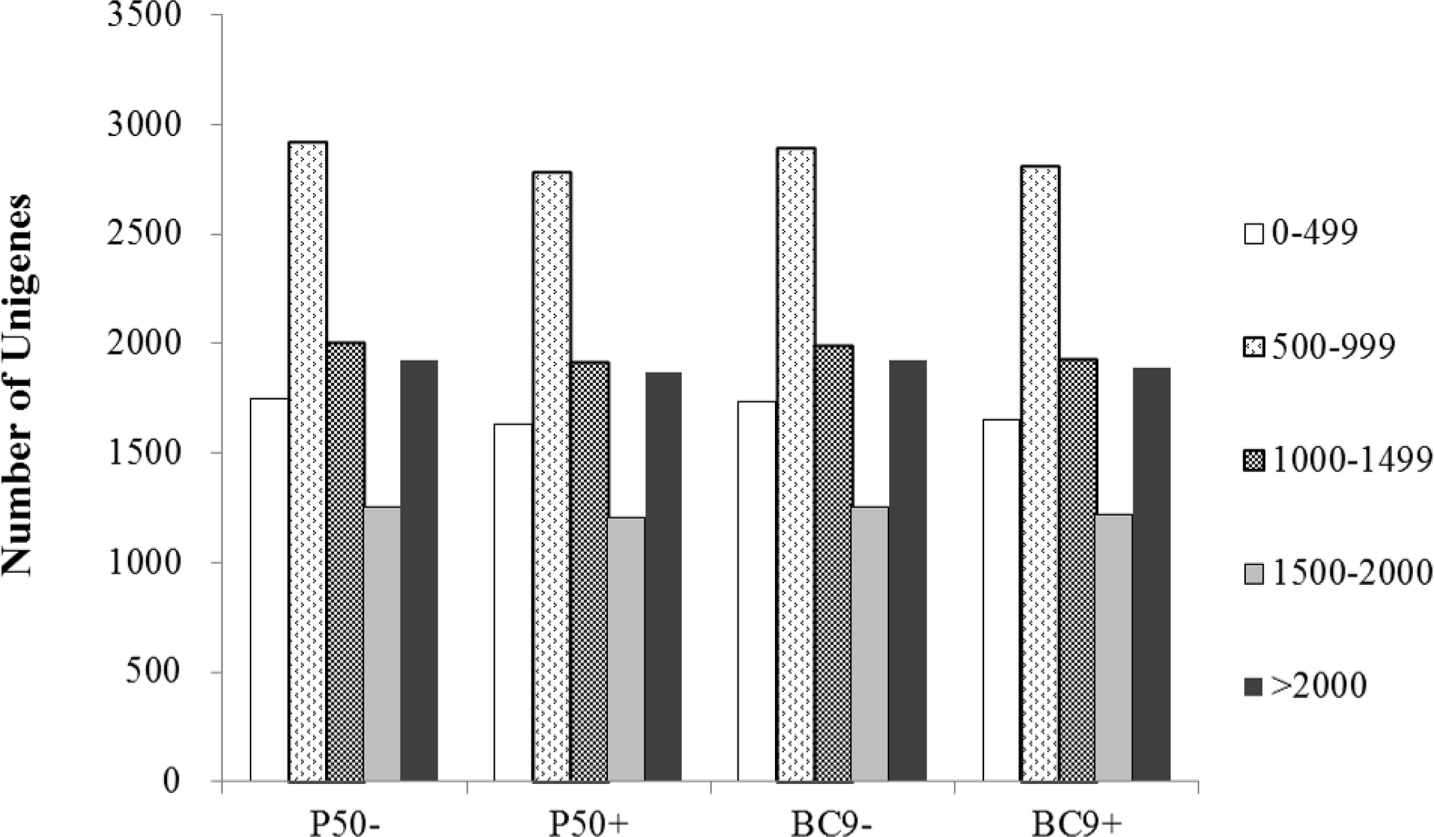
Length distribution of unigenes in the assembled transcriptomes. The x axis represents different groups and treatments and the y axis shows the number of unigenes.

Transcriptome sequencing is an efficient technology for comparing gene expression levels in different samples, and in our study, it was used to search and analyze DEGs among P50, BC9 control and treatment groups. A total of four cDNA libraries were sequenced: P50- (P50 treated with sterile water), P50+ (P50 infected with BmNPV), BC9- (BC9 treated with sterile water), BC9+ (BC9 infected with BmNPV), with each group created in triplicate. After removing the adaptors and low quality sequences, 144,439,382 sequence reads were obtained (Table 3). The GC content of each of the four libraries was approximately 50%, and CycleQ30% was greater than 89.91% for each library. Thus, the quality and accuracy of the sequencing data were sufficient for further analysis. Most of the reads matched silkworm genomic locations. All the unigenes matched previously described sequences with approximately 70% coverage. The length distribution of unigenes had similar patterns among the four libraries, suggesting that there was little bias in the construction of the four cDNA libraries (Fig 1).

**Table 3:** Summary statistics for silkworm genes based on the RNA-seq data.

	P50-	P50+	BC9-	BC9+
Total Reads	34,202,992	39,598,483	33,696,273	36,941,634
GC Content (%)	48	49	48	48
% ≥ Q30 (%)	91.42	90.74	90.07	90.28
Mapped Reads	27,261,542	31,333,514	26,640,096	29,131,867
Mapped Ratio (%)	79.72	79.08	79.06	78.89
Unique Mapped Reads	23,563,245	26,329,862	23,325,074	25,650,914
Unique Mapped Ratio (%)	68.90	66.51	69.21	69.47

### Unigenes annotation and classification

In order to annotate the unigenes, reference sequences were searched using BLASTX against the NCBI nr database (E-value < 10^−5^). A total of 12,591 of 13,342 unigenes provided a BLAST result (S1 Table). S2 Table shows the species with the closest match for each unigene. Most of the annotated sequences had the greatest homology with other *B*. *mori* sequences (87%), followed by *Danaus plexippus* (9%).

### RT-qPCR validation of differentially expressed transcripts

**Fig 2:**
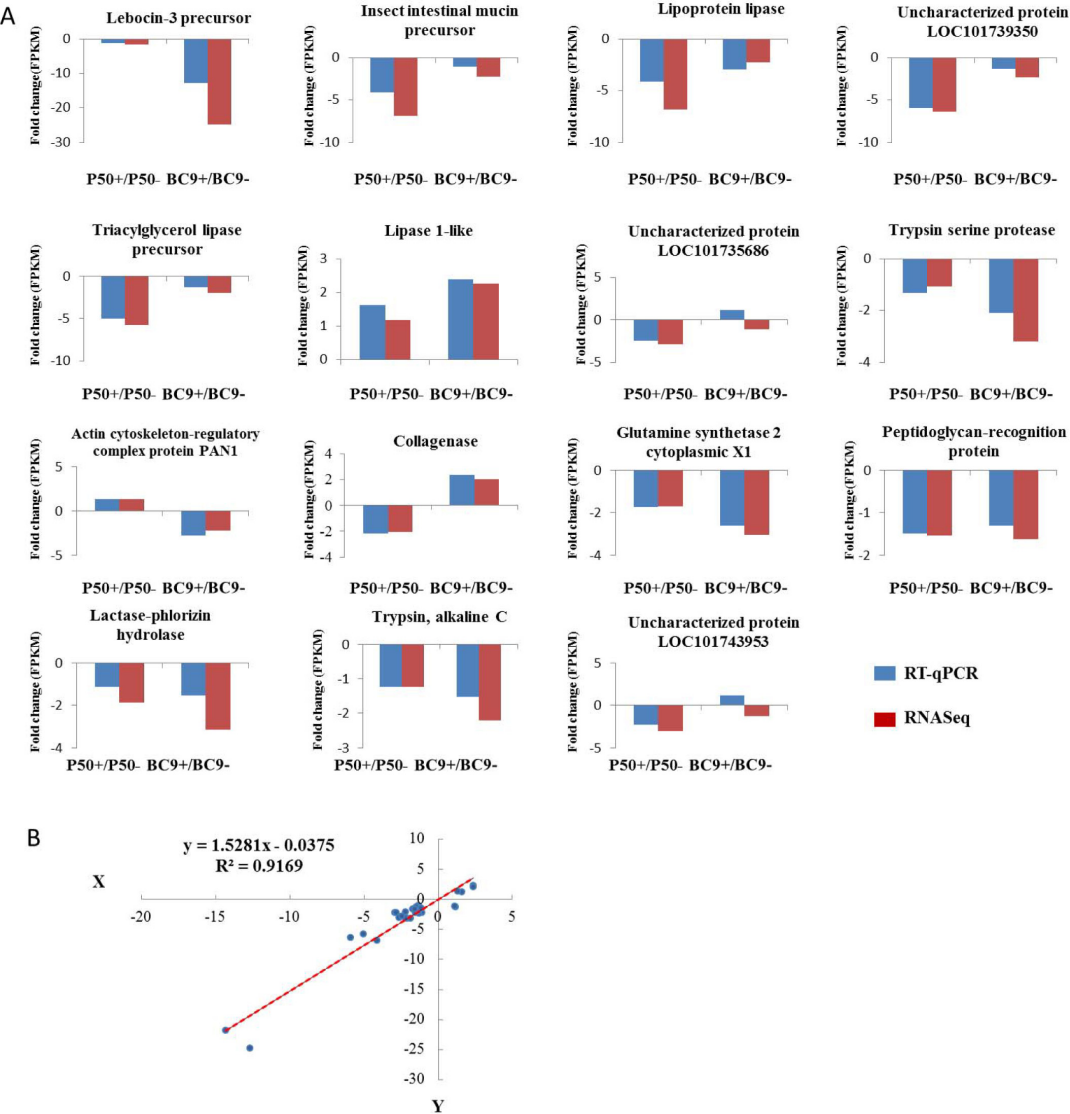
Correlation between gene expression ratios obtained from transcriptome data and RT-qPCR. (A) Expression ratios (FPKM fold change) obtained from transcriptome data (red) and RT-qPCR (blue). (B) Lineage analysis between the transcriptome and RT-qPCR. The ratios obtained by RT-qPCR (x-axis) were plotted against the ratios obtained by RNA-Seq (y-axis).

In order to determine the reliability of the transcriptome sequencing, the relative expression levels of 15 randomly selected genes were analyzed by RT-qPCR (Fig 2A). The results were consistent with the transcriptome data. For example, the gene encoding peptidoglycan-recognition protein was down-regulated in both RNA-seq and RT-qPCR analyses, with a similar fold change. The *lipase 1* gene was significantly up-regulated in the resistant strain BC9 after infection with BmNPV, which was also consistent with previously reported results [10]. Linear regression analysis of the correlation between RT-qPCR and RAN-seq (Fig 2B) showed an R^2^ (goodness of fit) value of 0.9169 and a corresponding slope of 1.5281, suggesting a strong positive correlation between RT-qPCR and transcriptome data. Therefore, the transcriptome data were satisfied for further analysis.

### Differentially expressed genes (DEGs) and their possible roles in host response to BmNPV

**Fig 3:**
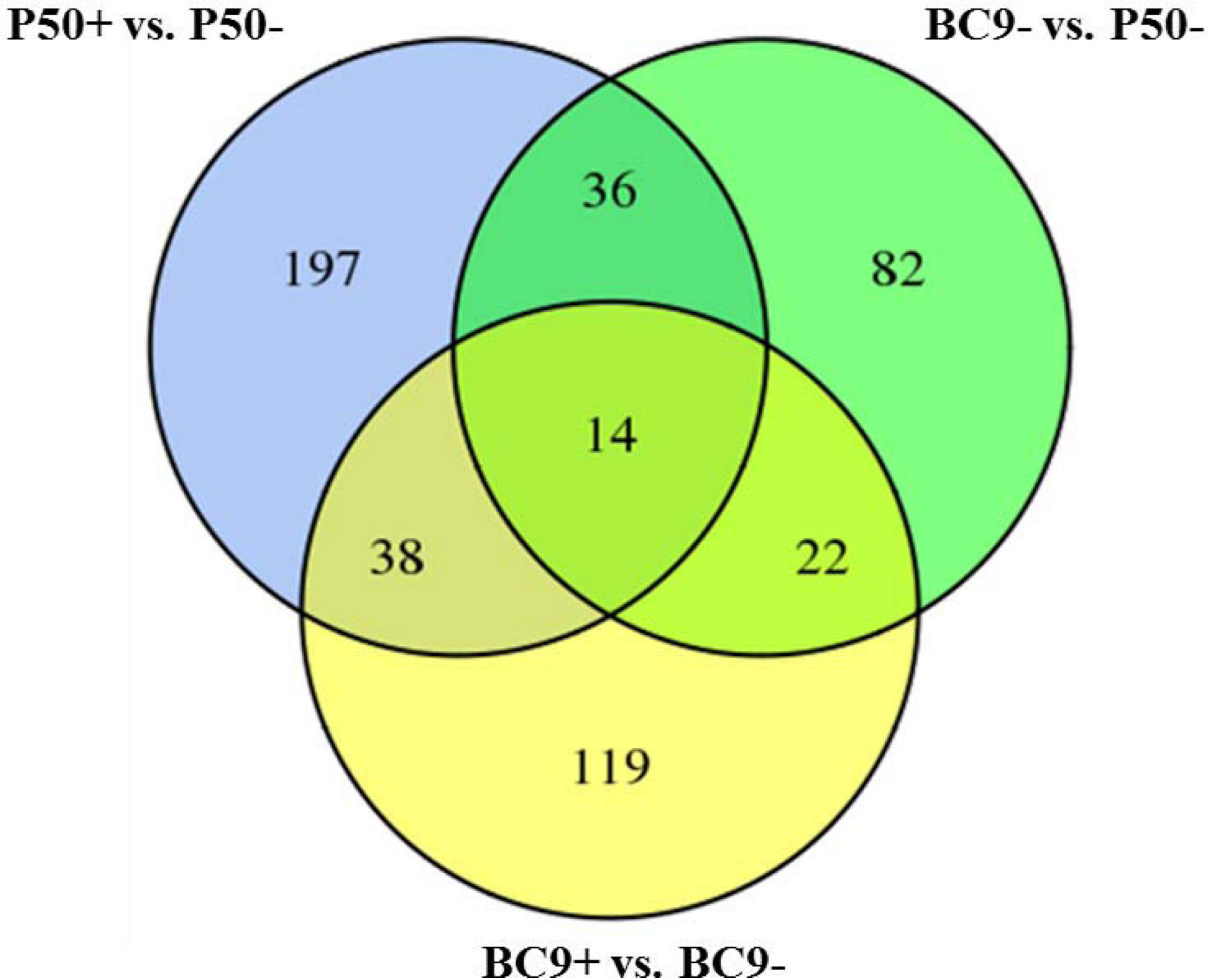
Venn diagram showing the DEGs related to BmNPV infection in different resistant strains.

To further elucidate which DEGs had a potential role in antiviral response, a Venn diagram was constructed. A total of 285 DEGs were found to be differentially regulated when comparing P50+ and P50-, of which 122 were up-regulated and 163 were down-regulated. Similarly, 193 DEGs were found to be differentially regulated in the comparison of BC9+ vs. BC9-, with 56 genes up-regulated and 137 down-regulated. In addition, there were 154 DEGs differentially regulated in the comparison of BC9- and P50-, among which 78 genes were up-regulated and 76 genes were down-regulated (Fig 3, Table 4, S3 Table).

**Table 4:** The number of up-regulated, down-regulated and unique DEGs of the pairwise comparisons of the experimental groups. Shared numbers of DEGs are also shown in Fig 3.

Groups	Total	Up-regulated genes	Down-regulated genes	Unique genes
**P50+ vs. P50-**	285	122 (43%)	163 (57%)	197
**BC9+ vs. BC9-**	193	56 (29%)	137 (71%)	119
**BC9- vs. P50-**	154	78 (51%)	76 (49%)	82

**Fig 4:**
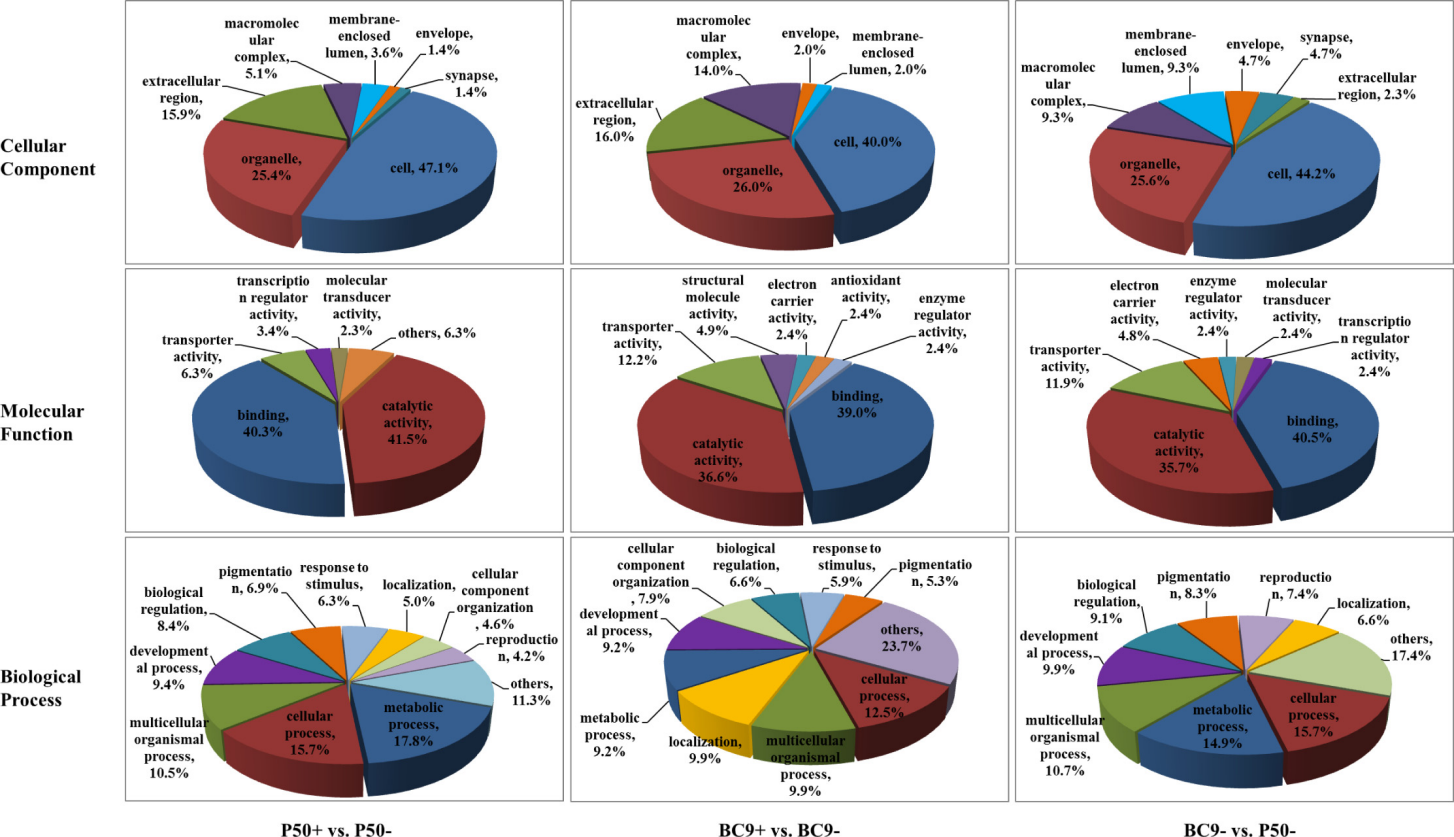
Gene ontology (GO) analysis of DEGs in different comparable groups. These genes were divided into groups based on cellular component, molecular function, and biological process. The percent of DEGs that could be assigned to the different categories are indicated.

There were 197, 119, 82 unique DEGs in P50+ vs. P50-, BC9+ vs. BC9- and BC9- vs. P50-, respectively. GO assignments were used to assign a functional classification to these DEGs. For cellular components, the number of unique DEGs fell into the macromolecular complex classification was distinct in BC9 and P50 following BmNPV infection. For molecular functions, the number of transporter activity related unique DEGs was distinct in BC9 and P50 following BmNPV infection. For biological progresses, the number of unique DEGs involved in metabolism processes and localization was distinct in BC9 and P50 following BmNPV infection (Fig 4).

### Analysis of DEGs associated with protein metabolism, cytoskeleton, and apoptosis

The comparisons of BC9+ vs. BC9- and P50+ vs. P50- identified many DEGs that might either be involved in silkworm defense against BmNPV or facilitate BmNPV infection. These genes could be divided into three categories: protein metabolism, cytoskeleton, and apoptosis.

Most of the DEGs (70%) associated with protein metabolism were down-regulated in BC9 following BmNPV infection. In contrast, 80% of the genes in P50 were up-regulated after BmNPV infection. Most of the DEGs (87.5%) associated with the cytoskeleton were up-regulated in P50 after BmNPV infection, but the number of up-regulated genes (50%) decreased in BC9. A total of 19 DEGs associated with apoptosis were identified following BmNPV infection. Ten of these DEGs were altered in BC9 after BmNPV infection. The other DEGs were all over-expressed in P50 following BmNPV infection (Table 5).

**Table 5:** DEGs involved in protein metabolism, cytoskeleton, and apoptosis after BmNPV infection in different resistant strains.

Name	Gene ID	P50- FRKM	P50+ FPKM	BC9- FPKM	BC9+ FPKM	P50+ vs. P50- ratio	BC9+ vs. BC9- ratio
**Protein metabolism**							
Hypothetical protein KGM_08787	BGIBMGA003894	6.479	7.031	6.991	4.125	1.085	0.590
B(0,+)-type amino acid transporter 1	BGIBMGA007713	34.455	33.024	25.861	18.795	0.958	0.727
L-asparaginase	BGIBMGA012995	21.645	24.361	21.849	16.429	1.125	0.752
NEDD8-conjugating enzyme UBE2F	BGIBMGA013486	8.256	7.871	7.639	10.033	0.953	1.313
4-aminobutyrate aminotransferase	BGIBMGA006823	106.219	101.463	117.167	155.906	0.955	1.331
Uncharacterized protein LOC101742492	BGIBMGA006234	21.208	16.606	15.485	14.839	0.783	0.958
Proton-coupled amino acid transporter 4	BGIBMGA001151	1.715	3.412	2.198	1.239	1.990	0.564
Y+L amino acid transporter 2	BGIBMGA010801	1.541	2.816	2.030	1.249	1.827	0.615
Solute carrier family 12 member 6	BGIBMGA003629	1.068	1.736	0.867	0.564	1.625	0.651
Cystathionine gamma-lyase	BGIBMGA003656	219.509	270.108	184.300	138.484	1.231	0.751
**Cytoskeleton**							
Actin	BGIBMGA013945	945.736	1115.438	637.646	1057.763	1.179	1.659
Muscle LIM protein isoform 1	BGIBMGA001202	124.039	124.062	89.846	127.161	1.000	1.415
Apolipophorins isoform X2	BGIBMGA013341	2.345	3.642	2.198	2.856	1.553	1.300
Putative villin	BGIBMGA003119	6.824	6.585	7.700	9.502	0.965	1.234
Zinc finger protein Gfi-1b	BGIBMGA006132	12.448	18.952	12.387	9.220	1.522	0.744
Actin cytoskeleton-regulatory complex protein PAN1	BGIBMGA004121	83.016	99.504	54.242	28.428	1.199	0.524
Actin cytoskeleton-regulatory complex protein PAN1	BGIBMGA004002	4834.384	6342.360	4000.747	1781.384	1.312	0.445
Actin cytoskeleton-regulatory complex protein PAN1	BGIBMGA010768	7.855	45.723	7.668	0.250	5.821	0.033
Proteasomal ATPase-associated factor 1	BGIBMGA003545	4.920	8.102	6.756	7.861	1.647	1.164
Actin-binding protein	BGIBMGA013080	2.026	3.810	2.813	3.095	1.880	1.100
ATPase family AAA domain-containing protein 3	BGIBMGA000542	23.055	17.196	20.730	21.882	0.746	1.056
**Apoptosis**							
Conventional protein kinase C	BGIBMGA014132	0.352	0.305	0.384	0.250	0.866	0.652
Pyruvate dehydrogenase kinase	BGIBMGA003258	6.211	5.928	4.519	3.213	0.955	0.711
P53	BGIBMGA013185	0.714	0.687	0.675	0.552	0.963	0.818
Creb	BGIBMGA006865	21.657	21.099	23.179	18.966	0.974	0.818
Cytochrome c	BGIBMGA009012	1149.891	1072.848	1153.371	1464.034	0.933	1.269
Cell death activator CIDE-B	BGIBMGA011008	4.881	4.655	4.116	6.451	0.954	1.567
Caspase Nc	BGIBMGA002841	5.533	6.677	6.469	6.021	1.207	0.931
cAMP-dependent protein kinase C1	BGIBMGA011429	17.894	23.160	20.027	21.581	1.294	1.078
Tak1	BGIBMGA010752	6.565	8.039	6.933	7.573	1.224	1.092
Apoptosis-inducing factor	BGIBMGA014381	0.836	1.080	0.633	0.725	1.291	1.145
Protein kinase ASK1	BGIBMGA010545	1.928	2.558	2.321	2.588	1.327	1.115
Ribosomal protein S6 kinase, 90 kda	BGIBMGA011088	14.457	19.178	17.556	20.070	1.327	1.143
Daxx	BGIBMGA007470	7.205	9.653	9.246	9.842	1.340	1.065
TRAF6	BGIBMGA001290	1.493	2.077	1.604	1.906	1.392	1.188
TNFSF5	BGIBMGA003585	0.257	0.206	0.376	0.577	0.799	1.535
Survivin 2	BGIBMGA003946	0.521	0.387	0.585	1.015	0.743	1.737
App	BGIBMGA008317	0.075	0.032	0.045	0.093	0.433	2.059
Buffy	BGIBMGA001845	0.261	0.047	0.000	0.000	0.181	NA

The ratio represents the fold change of FPKM values after infection with BmNPV: a ratio ≥ 1.2 indicates genes that were up-regulated, a ratio ≤ 0.83 indicates genes that were down-regulated. Abbreviation: na, not applicable.

### Alteration in immunity related gene expression after BmNPV infection in different resistant strains

Pathogen infection stimulated both cellular and humoral responses of insects [33–36]. Genes participating in innate immunity pathways were identified and analyzed in regards to their potential role in BmNPV infection (Table 6). Thirty DEGs were identified, which could be classified into Toll pathway, Imd pathway, polyphenol oxidase (PPO) pathway, pattern recognition receptor, and antimicrobial peptide. As shown in Table 6, 14 (47%) of these genes were down-regulated and 9 (30%) were up-regulated in BC9 after infection with BmNPV, while 17 (57%) were down-regulated and 6 (20%) were up-regulated in P50 after BmNPV infection.

**Table 6:** Relative FPKMs of innate immune-related genes in silkworm after BmNPV infection in different resistant strains.

Description	Gene ID	P50- FRKM	P50+ FPKM	BC9- FPKM	BC9+ FPKM	P50+ vs. P50- ratio	BC9+ vs. BC9- ratio
**Toll pathway**							
Protein toll-like	BGIBMGA011084	0.016	0.035	0.008	0.074	2.142	9.010
18 wheeler	BGIBMGA011037	0.048	0.111	0.089	0.124	2.314	1.396
Protein toll-like	BGIBMGA011034	1.936	2.607	1.903	1.829	1.347	0.962
Slit homolog 2 protein	BGIBMGA011085	0.049	0.000	0.040	0.008	0.000	0.212
Hypothetical protein KGM_16873	BGIBMGA011216	30.386	28.239	23.694	25.300	0.929	1.068
Toll-like receptor 13	BGIBMGA008840	0.572	0.842	0.645	0.307	1.472	0.475
Slit homolog 2 protein	BGIBMGA011025	0.112	0.128	0.061	0.070	1.144	1.157
Slit homolog 3 protein	BGIBMGA011082	1.351	1.454	1.333	1.952	1.076	1.464
Protein toll-like	BGIBMGA006244	0.182	0.207	0.226	0.093	1.139	0.412
**Imd pathway**							
Inhibitor of nuclear factor kappa-B kinase subunit beta	BGIBMGA008389	2.825	3.434	3.333	3.683	1.216	1.105
Nuclear factor NF-kappa-B p110 subunit isoform 1	BGIBMGA002465	19.945	17.731	23.296	17.230	0.889	0.740
Nuclear factor NF-kappa-B p110 subunit isoform 1	BGIBMGA002464	41.624	37.288	44.316	36.213	0.896	0.817
**PPO pathway**							
Serine protease inhibitor dipetalogastin	BGIBMGA009047	54.598	68.729	50.136	52.770	1.259	1.053
Serine protease inhibitor 6	BGIBMGA007729	0.372	0.858	0.549	0.350	2.308	0.638
**Pattern recognition receptor**							
C-type lectin 10	BGIBMGA006768	1.300	3.213	1.591	1.503	2.472	0.945
Macrophage mannose receptor 1	BGIBMGA002288	0.133	0.392	0.000	0.066	2.940	na
Beta-1,3-glucan recognition protein 2	BGIBMGA011609	12.509	7.548	33.869	7.289	0.603	0.215
Peptidoglycan-recognition protein SC2	BGIBMGA000584	3.019	4.118	3.421	3.479	1.364	1.017
Peptidoglycan recognition protein S6	BGIBMGA012866	0.722	0.253	0.734	0.081	0.351	0.110
**Antimicrobial peptide**							
Attacin	BGIBMGA002739	0.193	0.000	0.098	0.048	0.000	0.495
Attacin precursor	BGIBMGA002747	1.055	0.974	0.820	0.145	0.923	0.177
Cecropin-A precursor	BGIBMGA006280	0.481	0.000	0.328	0.325	0.000	0.989
Defense protein precursor	BGIBMGA014360	0.162	8.504	4.234	0.200	52.588	0.047
Gloverin 1 precursor	BGIBMGA013863	1.805	1.326	6.944	0.684	0.735	0.098
Gloverin 2 precursor	BGIBMGA005658	17.719	9.138	14.492	3.872	0.516	0.267
Gloverin 3 precursor	BGIBMGA013803	3.068	2.236	1.915	1.245	0.729	0.650
Hemolin	BGIBMGA008736	0.000	0.022	52.634	0.136	na	0.003
Lebocin-3precursor	BGIBMGA006775	4.279	2.530	26.723	0.912	0.591	0.034
Lysozyme	BGIBMGA007458	1.936	4.622	0.876	2.108	2.387	2.407
Moricin 1	BGIBMGA011495	0.000	0.000	0.379	0.192	na	0.507

The ratio represents the fold change in the FPKM value after infection with BmNPV; a ratio ≥ 1.2 indicates genes that were up-regulated, a ratio ≤ 0.83 indicates genes that were down-regulated. Abbreviation: na, not applicable.

### Role of selected DEGs in BmNPV resistance

**Fig 5:**
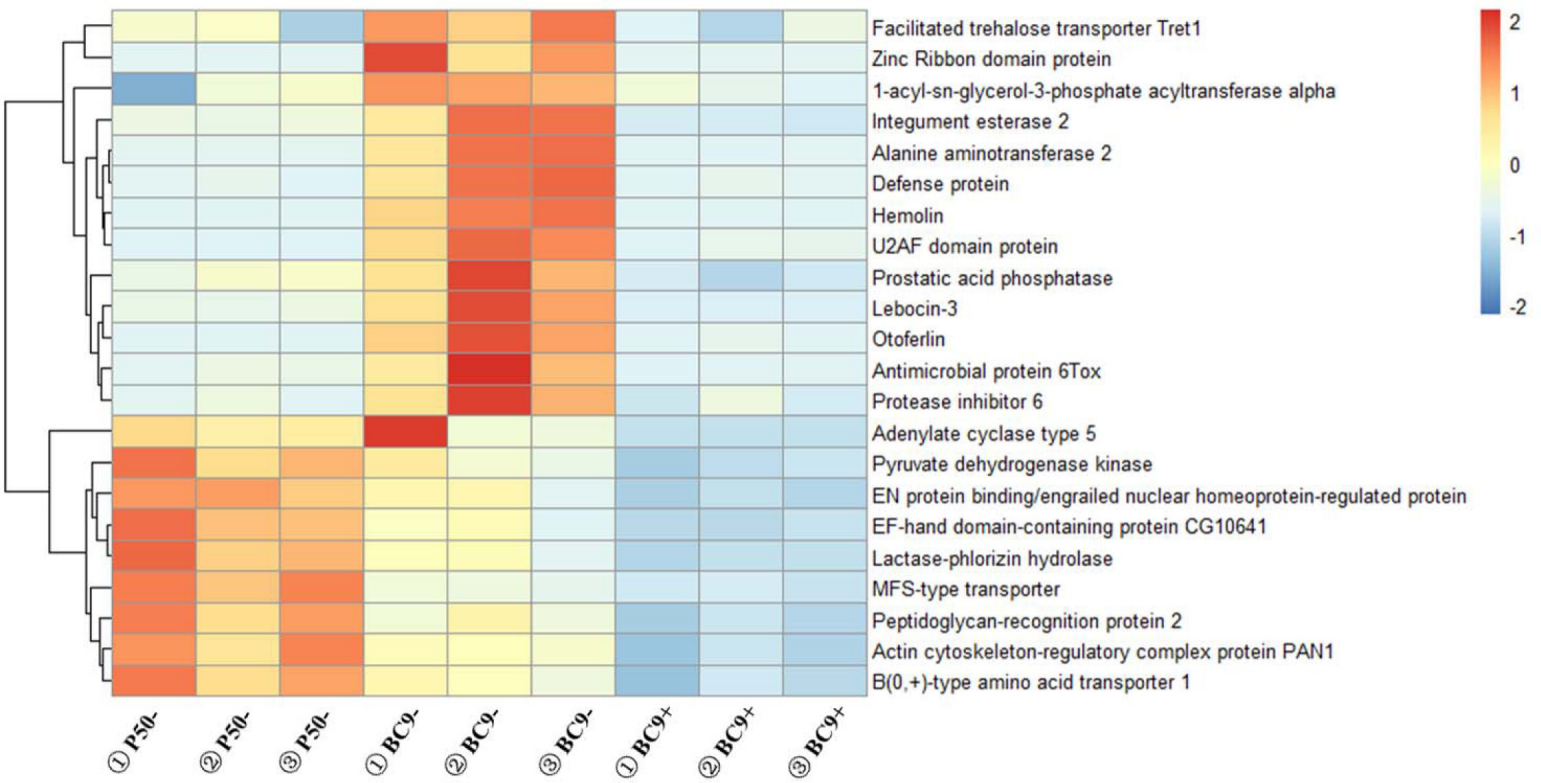
Expression patterns of selected genes related to BmNPV resistance in different resistant strains. Each row represents a different gene, with blue, yellow and red indicating low, medium and high levels of gene expression, respectively.

**Fig 6:**
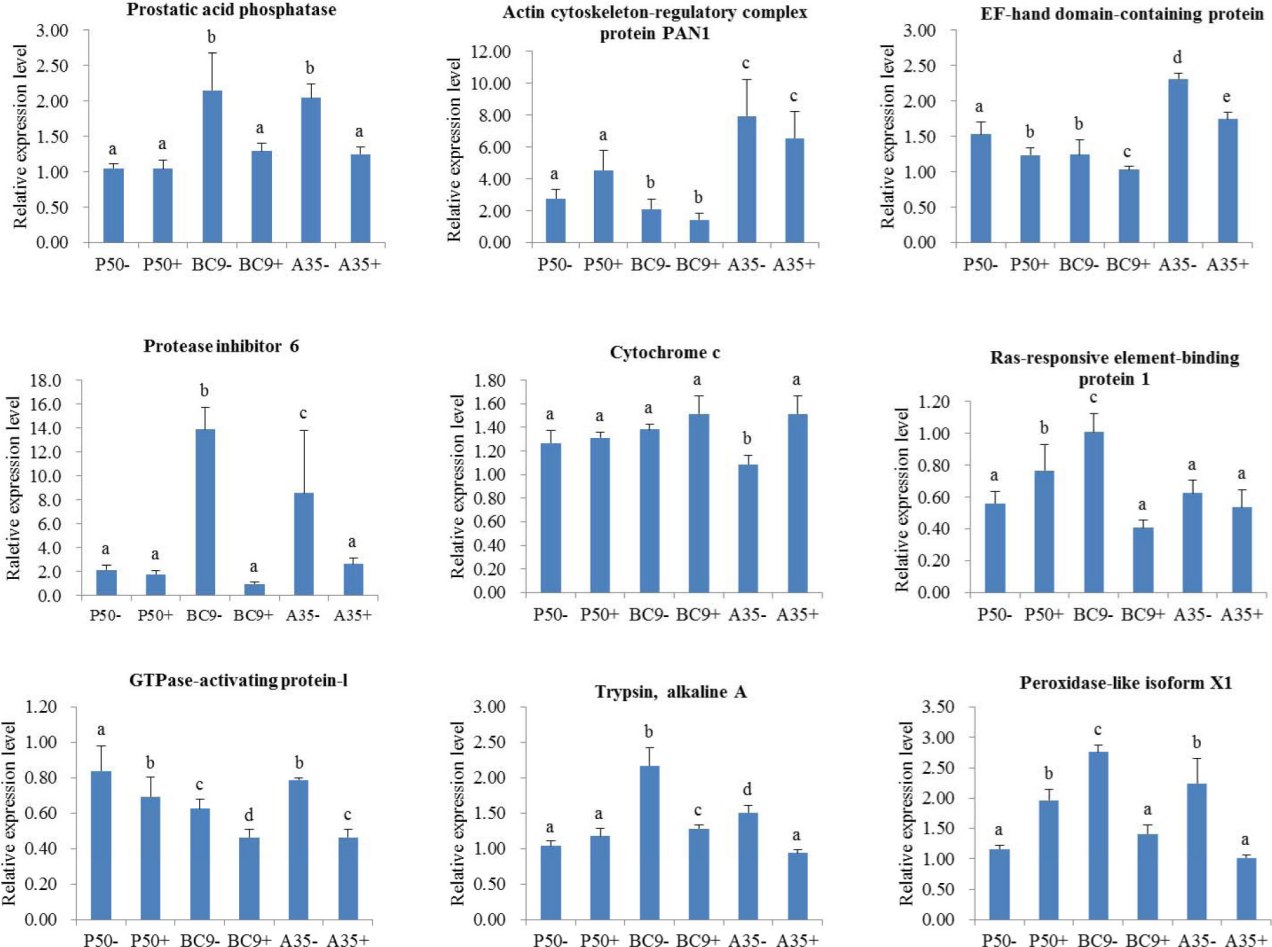
Real-time PCR analysis of expression profiles of several genes in *B*. *mori* midgut. After removing genetic background and individual immune stress response genes, 22 differentially expressed genes of interest potentially involved in resistance to NPV infection were obtained. Additionally, 119 genes unique differentially expressed in the isogenic-line BC9 (resistant strain) following BmNPV infection were observed. In order to further conformed the function of these genes, 4 genes from 22 DEGs and 5 genes from 119 DEGs with well reported previously were selected to conduct RT-qPCR.

After removing the genetic background and strain specific immune stress response genes, a total of 22 DEGs were identified as possibly being involved in silkworm resistance to BmNPV (Fig 3, Table 7). For BC9- vs. P50-, 13 genes were up-regulated and the rest were down-regulated. However, the 22 genes were all down-regulated in BC9 following BmNPV infection (Fig 5). Some genes, including prostatic acid phosphatase, protease inhibitor 6, actin cytoskeleton-regulatory complex protein PAN1, and EF-hand domain-containing protein, were further analyzed by RT-qPCR (Fig 6). After BmNPV infection, the expression levels of 4 genes were down-regulation in BC9 and A35 (resistant strain) (Fig 6), which was consistent with the transcriptome data. Thus, we deduced that the 22 genes were possibly involved in resistance to BmNPV infection. Gene functions fell into the following categories: transport, virus replication, intracellular innate immunity, and apoptosis.

**Table 7:** Relative FPKMs of 22 DEGs of interest in BC9 following BmNPV infection.

Name	Gene ID	P50- FPKM	P50+ FPKM	BC9- FPKM	BC9+ FPKM	BC9- vs. P50- FPKM	BC9+_vs._BC9- FPKM
Peptidoglycan-recognition protein 2	BGIBMGA000583	300.13	229.37	197.96	121.57	0.66	0.61
Adenylate cyclase type 5	BGIBMGA002064	3.52	2.39	3.48	1.14	0.99	0.33
Pyruvate dehydrogenase kinase	BGIBMGA003258	6.21	5.93	4.52	3.21	0.73	0.71
MFS-type transporter	BGIBMGA003409	50.65	47.67	15.50	6.82	0.31	0.44
Actin cytoskeleton-regulatory complex protein PAN1	BGIBMGA004121	83.02	99.50	54.24	28.43	0.65	0.52
B(0,+)-type amino acid transporter 1	BGIBMGA007713	34.46	33.02	25.86	18.79	0.75	0.73
EF-hand domain-containing protein CG10641	BGIBMGA008867	36.74	28.33	25.33	18.58	0.69	0.73
Lactase-phlorizin hydrolase	BGIBMGA010811	1751.88	1141.90	913.76	411.25	0.52	0.45
EN protein binding/engrailed nuclear homeoprotein-regulated protein	BGIBMGA011701	49.08	54.49	37.03	27.34	0.75	0.74
Antimicrobial protein 6Tox	BGIBMGA000861	0.59	0.37	3.91	0.26	6.57	0.07
Facilitated trehalose transporter Tret1	BGIBMGA004426	3.90	6.88	9.06	3.19	2.32	0.35
Protease inhibitor 6	BGIBMGA004869	7.51	6.49	51.58	4.39	6.86	0.09
Lebocin-3	BGIBMGA006775	4.28	2.53	26.72	0.91	6.25	0.03
1-acyl-sn-glycerol-3-phosphate acyltransferase alpha	BGIBMGA007880	7.70	2.58	17.19	8.46	2.23	0.49
Hemolin	BGIBMGA008736	0.00	0.02	52.63	0.14	NA	0.00
Integument esterase 2	BGIBMGA009544	2.39	2.49	8.48	1.12	3.55	0.13
Alanine aminotransferase 2	BGIBMGA011984	0.34	0.13	9.50	0.13	27.59	0.01
Otoferlin	BGIBMGA012258	0.02	0.04	2.23	0.05	96.93	0.02
U2AF function domain protein	BGIBMGA013991	0.00	0.07	2.95	0.15	NA	0.05
Defense protein precursor	BGIBMGA014360	0.16	8.50	4.23	0.20	26.18	0.05
Prostatic acid phosphatase	BGIBMGA014369	71.98	60.03	111.41	54.97	1.55	0.49
Zinc Ribbon domain protein	NewGene_1603	0.00	0.00	2.17	0.00	NA	0.00

The ratio represents the fold change in the FPKM value after infection with BmNPV; a ratio ≥ 1.2 indicates genes that were up-regulated, a ratio ≤ 0.83 indicates genes that were down-regulated. Abbreviation: na, not applicable.

## Discussion

Despite the confirmation of an association between many genes and proteins and resistance to BmNPV, the molecular mechanism of antiviral activities was still unclear. Here, transcriptome sequencing was carried out to identify genes related to BmNPV-resistance in silkworm across the genome. By using the near-isogenic line BC9 (resistant strain) and the recurrent parent P50 (susceptible strain) to study silkworm antiviral mechanisms, some DEGs responding to BmNPV infection were successfully identified after comparing infected groups and controls in the two strains.

### Overview of specific DEGs in two strains following BmNPV infection

Based on the GO analysis, more DEGs were found to be involved in metabolic processes in BC9 (17.8%) than in P50 (9.2%) following BmNPV infection (Fig 4), which was consistent with previous reports for *Sogatella furcifera* (Hemiptera: Delphacidae) and *Campoletis sonorensis* (Hymenoptera: Ichneumonidae) [35, 36]. Approximately twice as many DEGS related to transporter activity were identified in BC9 (12.2%) than in P50 (6.3%) (Fig 4). Down-regulation of transporter related genes, such as lactase-phlorizin hydrolase (LPH), B(0,+)-type amino acid transporter 1 (BAT1), actin cytoskeleton-regulatory complex protein PAN1 (PAN1), MFS-type transporter (MFS), and otoferlin, could repress virus infection in host cells [37–42]. Therefore, the increased number of these genes with altered expression levels in BC9 might be related to BmNPV infection. Moreover, the number of macromolecular complex genes in BC9 (14%) following BmNPV infection that showed an increase in expression compared with P50 (5.1%) were similar to previous reports [43, 44].

### Protein metabolism, cytoskeleton, and apoptosis may play an important role in host response to BmNPV infection

Cellular and metabolic processes will be dramatically changed after viral infection [35]. In our study, several DEGs that participate in protein metabolism were found to be of interest. For example, solute carrier family 12 is involved in transporting extraordinarily diverse solutes [45], and cystathionine gamma-lyase participates in hydrolysis of cystathionine [46]. Viruses may have to rely on cell proteins to accomplish replication in intercellular regions [47], therefore, the down-regulation of the genes involved in protein metabolism could inhibit the replication of BmNPV in the host cell. We speculated that the down-regulation of these genes affected virus replication.

Cytoskeleton-dependent intracellular transport is an important strategy for transport of viral particles to different destinations [48]. In this study, some cytoskeleton related genes were found to be of interest, including actin cytoskeleton-regulatory complex protein PAN1 and actin-binding protein. These genes related to actin-coupled endocytosis could promote viral transport [49, 50]. We speculated that the down-regulation of the cytoskeleton genes might affect BmNPV transport.

Apoptosis plays a vital role in regulating cell response in Lepidopteran insects during viral infections, where larvae use selective apoptosis and subsequent sloughing of the infected cells in the midgut epithelium to resist virus infection [51, 52]. In this study, some genes related to activation of apoptosis were found to be of interest, including cytochrome c, inhibitor of caspase-activated DNase, amyloid precursor protein and B-cell lymphoma protein 2. The activity of caspase-activated DNase was blocked by Hepatitis C virus core at physiological levels, resulting in the inhibition of apoptotic cell death [53]. Amyloid precursor protein is a member of several signaling pathways that are involved in abnormal cell cycles, subsequently leading to apoptosis [54]. B-cell lymphoma protein 2 could bind to BH3 domains of various pro-apoptotic regulators to activate apoptosis [55]. The overexpression of cytochrome c in rabies virus showed a decreased pathogenicity *in vitro* and *in vivo* [56]. Based on their role in apoptosis activation, hosts need to increase the expression level of these genes to promote apoptosis when exposed to a virus; this supposition explains the up-regulation of genes involved in apoptosis in the transcriptome following BmNPV infection. Additionally, a significantly higher relative transcriptional level of cytochrome c was detected by RT-qPCR in BC9 and A35 (Fig 6), which indicated the up-regulation of this gene could activate apoptosis to repress further BmNPV infection. However, some genes were down-regulated, including cysteine aspartic acid specific protease 9L, protein kinase A, apoptosis-inducing factor, apoptosis signal-regulating kinase 1, TNF-receptor-associated factor 6, TGF-beta-activated kinase 1, and p90 ribosomal S6 kinase. The inhibition of these genes may strongly impair viral infectivity and virus-induced apoptosis [57–62].

### Changes to immune gene expression following infection with BmNPV

Although some differential expression of immune genes was observed, this might not be considered biologically important due to low expression levels; low expression levels were also found after BmCPV infection [21]. In this study, most immune related genes were down-regulated, with a few exceptions, such as the 2-fold up-regulation of toll-like protein and lysozyme. The expression of several other genes, including 18 wheeler, macrophage mannose receptor 1, and slit homolog 3 protein, were also up-regulated during the BmNPV infection. Unfortunately, the relationship between immune genes and BmNPV remains unclear and requires further study. We presumed that the low expression levels of immunity related genes may be associated with the disruption of the immune system by BmNPV, similar to the pathogenicity of human immunodeficiency virus (HIV).

### Multiple genes have potential roles in repressing BmNPV infection

Based on the Venn diagram, 22 genes of interest were identified, all of which were down-regulated in BC9 following BmNPV infection. These genes were grouped based on their functions, as reported in the literature. These groups, including transport, virus replication, intracellular innate immune, and apoptosis, may play an important role in the process of silkworm resistance to BmNPV.

Several genes related to virus transport, including lactase-phlorizin hydrolase (LPH) [37], B(0,+)-type amino acid transporter 1 (BAT1) [38, 46], actin cytoskeleton-regulatory complex protein PAN1 (PAN1) [63], and otoferlin [41, 42] were identified in this study. The expression level of these genes were all down-regulated in BC9 following BmNPV infection (Fig 5). The resistant strain A35, a donor parent, was used to validate our results. The expression level of PAN1 was chosen for further testing by RT-qPCR. Expression levels of PAN1 were down-regulated in both BC9 and A35 following BmNPV infection (Fig 6). The down-regulation of virus transport-related genes could inhibit the transmembrane and intracellular transport of BmNPV thereby preventing further infection. MFS-type transporter (MFS) [39, 40], a transmembrane facilitator, was induced to increase intracellular monovalent ion concentrations, which led to lysis and cell death after HIV-1 infection. The down-regulation of this gene in silkworm could potentially block BmNPV infection.

Several genes related to virus replication were found to be of interest, including engrailed nuclear homeoprotein-regulated protein [64], 1-acyl-sn-glycerol-3-phosphate acyltransferase alpha (ASGPA) [65, 66], alanine aminotransferase 2 (ALT2) [67, 68], U2AF domain protein (U2AF) [69, 70], adenylate cyclase type 5 (ACT5) [71], EF-hand domain-containing protein (EFHP) [72, 73], prostatic acid phosphatase (PAP) [74], and zinc ribbon domain protein (ZRDP) [75]. In this study, the expression level of these genes were all down-regulated in the transcriptome of BC9 following BmNPV infection (Fig 5). In order to validate the results, RT-qPCR was conducted as described above. The expression levels of EFHP and PAP were lower following BmNPV infection in both BC9 and A35 (Fig 6). The down-regulation of these genes could directly or indirectly participate in repressing BmNPV replication in host cells.

Of the genes related to apoptosis, the expression level of pyruvate dehydrogenase kinase (PDK) was obviously inhibited after treatment with apoptosis-inducing agents [76]; such down-regulation might activate apoptosis in response to BmNPV infection.

Protease inhibitor 6 is a member of the protein superfamily that contains TIL functional domain. Zhao et al. used genome sequences to demonstrate that the expression level of the TIL superfamily were down-regulated following BmNPV infection [77], which was consistent with our results (Fig 6). Furthermore, peptidoglycan-recognition protein 2, hemolin, facilitated trehalose transporter Tret1, integument esterase 2, defense protein precursor, and antimicrobial protein 6Tox precursor also showed differential expression following BmNPV infection. The relationship of these genes to BmNPV is an important area for further study.

Four DEGs were down-regulated in BC9 following BmNPV infection, including ras-responsive element-binding protein 1, GTPase-activating protein, trypsin alkaline A and peroxidase (Fig 5). Previous studies revealed that these proteins play a role in virus infections. Mdv1-miR-M4 encoded by Marek's disease virus efficiently targeted the 3' untranslated regions of ras-responsive element-binding protein 1 (RREB1) [78]. TBC domain proteins belonging to a GTPase-activating protein were knocked out by double stranded RNA interference (RNAi), which led to a decrease in the level of transcripts of white spot syndrome virus genes [79]. Trypsin in the myocardium was able to trigger acute myocarditis following influenza A virus infection [80]. Overexpression and RNA silencing studies revealed that peroxidase was involved in the production of hepatitis C virus particles [81]. Moreover, RT-qPCR results indicated that the expression levels of all genes were down-regulated in BC9 and A35 following BmNPV infection (Fig 6). Therefore, we speculated that the down-regulation of these genes might be involved resistance to BmNPV infection.

### Hypothesized modal analysis of the roles of the screened DEGs in silkworm resistance to BmNPV infection pathway

**Fig 7:**
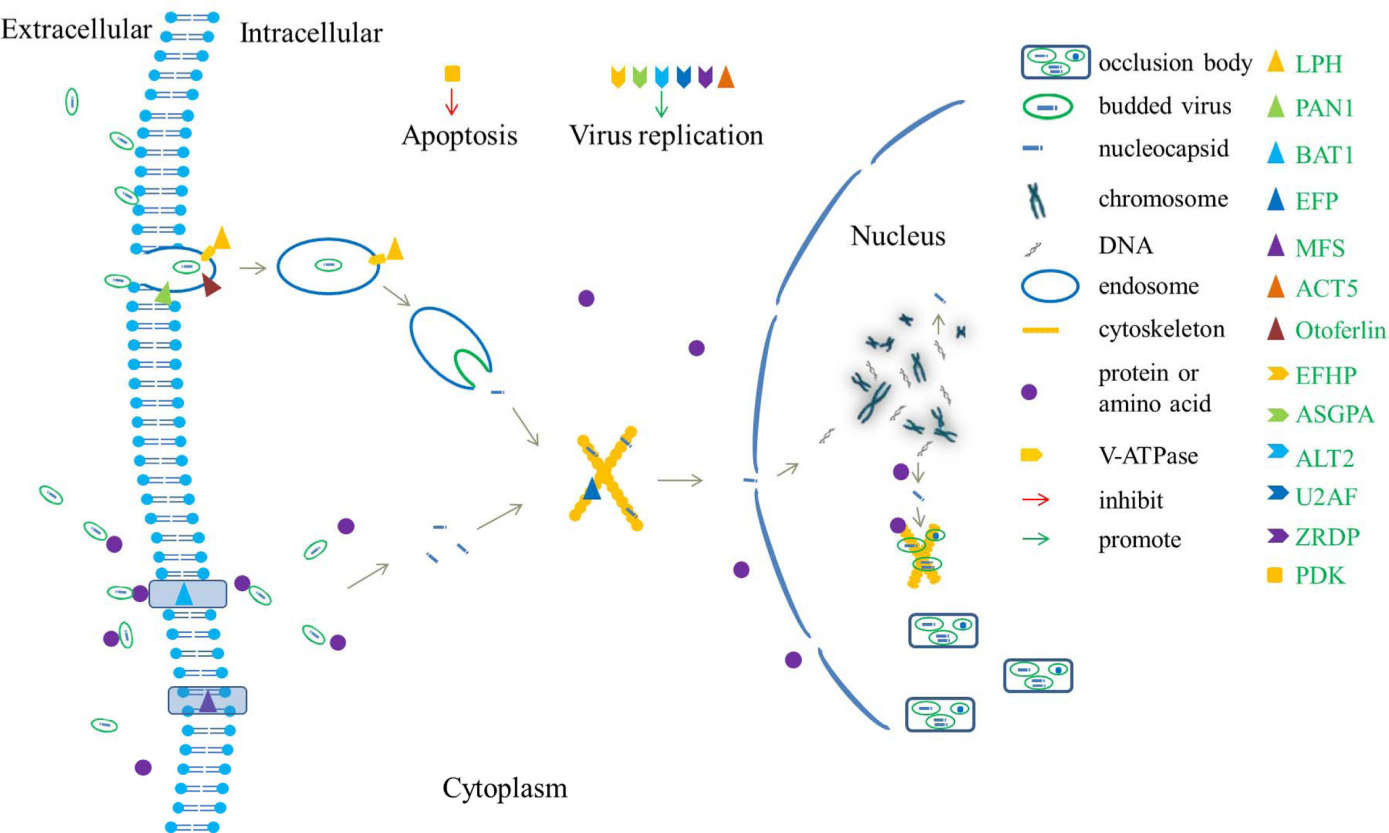
Hypothesized modal analysis of the roles of the screened DEGs in BmNPV infection pathway. V-ATPase is activated by LPH to promote viral entry into the cytoplasm, a process which was also effected by PAN1 and otoferlin. BAT1 related channel could serve as an alternative pathway for virus transmembrane transport. The released nucleocapsid is transported into the nucleus with the help of EFP. During replication, EFHP, ASGPA, ALT2, U2AF, ACT5 and ZRDP play an important role in facilitating virus replication. MFS is induced by the virus to increase cell volume leading to cell death. At the same time, the apoptosis process could be triggered by PDK to inhibit BmNPV infection.

We hypothesized that the 22 DEGs discussed above played a role in the process of host response to BmNPV infection. The endocytosis process is triggered when the BmNPV nucleocapsid containing envelope binds to the cytomembrane. Vacuolar ATP synthase is activated by LPH to promote the fusion of the envelope and endosome thereby releasing the nucleocapsid into the cytoplasm. This process can be promoted by PAN1 and otoferlin. However, the transmembrane transport channel is an alternative pathway for virus to enter the cytoplasm, a process which can be facilitated by BAT1. The released nucleocapsid is transported into the nucleus with the help of the cytoskeleton (EFP). Once viral DNA is released into the nucleus, it will utilize host nucleotides to complete replication. In the final step of replication, viral DNA has to rely on host cell amino acids for assembly on the cytoskeleton (Table 5) [82]. In the cytoplasm, EFHP, ASGPA, ALT2, U2AF, ACT5 and ZRDP play an important role in facilitating virus replication, although the exact mechanism is still unclear. Moreover, transmembrane protein, MFS, is induced by BmNPV to increase cell volume, leading to lysis and cell death. We speculated that the down-regulation of these genes may affect the entry of virus into host cells and virus replication. The apoptosis process could also be triggered by PDK to inhibit BmNPV from further infecting other cells once BmNPV entered a host cell (Fig 7).

Taken together, our results provide useful information on silkworm resistance to BmNPV infection.

## Supporting Information

S1 Table The annotation results for all unigenes. (XLSX)

S2 Table The species distribution for the BLAST matches. (XLSX)

S3 Table Genes that were differentially expressed in P50+ vs. P50-, BC9+ vs. BC9-, and BC9- vs. P50-. (XLSX)
